# ModEnzA: Accurate Identification of Metabolic Enzymes Using Function Specific Profile HMMs with Optimised Discrimination Threshold and Modified Emission Probabilities

**DOI:** 10.1155/2011/743782

**Published:** 2011-03-29

**Authors:** Dhwani K. Desai, Soumyadeep Nandi, Prashant K. Srivastava, Andrew M. Lynn

**Affiliations:** ^1^Biological Oceanography Division, Leibniz Institute of Marine Sciences (IFM-GEOMAR), Düsternbrooker Weg 20, 24105 Kiel, Germany; ^2^Department of Biochemistry, Microbiology and Immunology (BMI), Ottawa Institute of Systems Biology (OISB), Faculty of Medicine, University of Ottawa, Ottawa, ON, Canada K1H 8M5; ^3^Physiological Genomics and Medicine, MRC Clinical Sciences, Imperial College, London W12 0NN, UK; ^4^School of Computational and Integrative Sciences, Jawaharlal Nehru University, New Delhi 110067, India

## Abstract

Various enzyme identification protocols involving homology transfer by sequence-sequence or profile-sequence comparisons have been devised which utilise Swiss-Prot sequences associated with EC numbers as the training set. A profile HMM constructed for a particular EC number might select sequences which perform a different enzymatic function due to the presence of certain fold-specific residues which are conserved in enzymes sharing a common fold. We describe a protocol, ModEnzA (HMM-ModE Enzyme Annotation), which generates profile HMMs highly specific at a functional level as defined by the EC numbers by incorporating information from negative training sequences. We enrich the training dataset by mining sequences from the NCBI Non-Redundant database for increased sensitivity. We compare our method with other enzyme identification methods, both for assigning EC numbers to a genome as well as identifying protein sequences associated with an enzymatic activity. We report a sensitivity of 88% and specificity of 95% in identifying EC numbers and annotating enzymatic sequences from the E. coli genome which is higher than any other method. With the next-generation sequencing methods producing a huge amount of sequence data, the development and use of fully automated yet accurate protocols such as ModEnzA is warranted for rapid annotation of newly sequenced genomes and metagenomic sequences.

## 1. Introduction

Emergence of next-generation sequencing technologies [1] and complete genome sequencing projects have greatly facilitated the process of unraveling the full repertoire of biological functions that an organism possesses. For a pathogenic organism, such a compilation of functions has a direct implication in identifying potential drug targets, for example, selecting genes or functions which are unique to the pathogen and not present in the host organism. Metabolic enzymes have for long been considered as a promising group from which such drug targets can be identified [2]. Enzymes form a sizable part of the druggable genome. Druggability is equated with the presence of certain folds in the proteins which can favour interactions with drug-like chemical compounds. The active sites or ligand binding pockets of enzymes are prime examples of druggable folds such that around 47% of the small molecule drugs available in the market have an enzyme target [3, 4]. Enzyme drug targets have been identified and exploited in a number of pathogenic protozoans [5–7], bacteria such as *M. tuberculosis* [8, 9], and fungal pathogens such as *C. albicans* [10–12]. Metagenome projects resulting from recent advances in environmental shotgun sequencing also provide opportunities for metabolic enzyme mapping as well as studying the environmental impact on evolution of metabolism [13, 14].

Metabolic reconstruction is a process that aims to develop a complete overview of the metabolic capabilities of an organism from the genome or metagenome sequence. There are various databases to aid metabolic reconstruction which integrate and curate data from different sources, for example KEGG [15] which combines genomic, chemical, and network information, PUMA2 [16] which provides an array of tools for comparative genomics, and MetaCyc [17] which is a multiorganism pathway/genome database. Accurate identification of metabolic enzymes from a fully sequenced genome is a very important step towards such a reconstruction. The most common approach for detection of an enzyme function in a given genome is on the basis of sequence similarity with homologues whose function is known, which can be accomplished by using either sequence-sequence comparison methods such as BLAST [18] and FASTA [19] or profile-sequence comparison methods like PSI-BLAST [20] and HMMER [21]. Different protocols have been developed using these methods that detect enzymatic function at the level of the EC number. PRIAM [22], for example, generates position-specific scoring matrices for collections of sequences which are associated with the same EC number which are then used to score sequences in a genome using reverse positionspecific blast (RPS-BLAST) [23]. MetaSHARK [24] improves on the sensitivity of PRIAM enzyme prediction by using hidden Markov models to identify corresponding enzymes directly from the genomic sequence. Other approaches for enzymatic function inference include checking for the presence of a conserved pattern or motif and identification of functionally critical residues. EFICAz [25] is a protocol which combines these two approaches along with an iterative HMMER-based procedure for generating multiple alignments for EC number families and pairwise sequence comparison using a family-specific identity threshold. It has recently been extended by including additional components based on support vector machine (SVM) models [26].

Metabolic enzymes belonging to certain core pathways are conserved in all three domains of life (namely archaea, bacteria, and eukaryotes) and can thus be easily identified using sequence homology [27]. However, there are a total of 4905 unique EC number entries in the enzyme [28] database release of 19 January, 2010, out of which only 2507 entries have one or more sequence associated with them. This has led to development of various algorithms and methods which try to associate sequences with enzymatic activities hitherto unannotated in an organism (hence, a pathway “hole” [29]) and can be collectively termed as “hole-filling” algorithms. A variety of other information is used in addition to the sequences, for example topology of the metabolic network [30, 31] or genomic evidences such as chromosomal clustering of operons [32], a combination of both [29], or an ensemble of various kinds of methods such as gene coexpression, phylogenetic profile cooccurence, protein fusion, and so forth [33, 34]. Whereas these methods can be used in conjunction or as a complement to the profile-sequence comparison methods to obtain a complete overview of the metabolic reactions of an organism, the latter group of methods, nevertheless, still retains its importance. 

A profile HMM constructed using Swissprot sequences for a particular EC number might score sequences belonging to other EC groups very highly if the enzymatic activities have developed in the same protein fold and therefore share certain fold-specific residues [35]. For example, the Alpha/Beta hydrolase fold (SCOP [36] classification) has 35 protein families which span through various enzymatic functions in terms of EC numbers. A group of 9 EC numbers, all of which are carboxylic ester hydrolases (EC 3.1.1) with different substrate specificities, are included in this fold group (Supplementary Table ST1 available online at doi:10.1155/2011/743782) and would be expected to have common fold-specific signals which would be conserved in sequences belonging to these EC numbers. As a result, the profile HMM for the EC 3.1.1.8 used with default parameters selects sequences from 5 other EC groups (Supplementry Figure SF1, inset available online at doi:10.1155/2011/743782). Figure SF1 also depicts the overall structural similarity between representatives from each of the six EC groups. 

We have earlier described the use of negative training sequences (i.e., sequences of different functions related to the training sequences by virtue of sharing a common fold) to both optimise the discrimination threshold as well as modify the emission probabilities of the profile HMMs to increase its specificity. We have used relative entropy of the amino acid probabilities of the positive and negative training sequences to select residues in the positive alignment which are responsible for its specific function as opposed to residues which are similarly conserved in both the negative and positive training sequence sets [35]. In this paper, we describe ModEnzA (HMM-ModE Enzyme Annotation), where we apply HMM-ModE to create profile HMMs which are specific at the functional level as defined by the EC classification. We enrich the training set by mining sequences from the nonredundant (NR) database for increased coverage of the EC numbers and thus, increased sensitivity. We use the Markov clustering algorithm (MCL) [37] to partition the EC sequence sets into clusters corresponding to nonorthologous sequences or oligomeric subunits. The ModEnzA protocol is used to annotate metabolic enzymes from completely sequenced reference genomes. We present a comparative analysis of our protocol with other methods used for genome-wide enzyme identification such as PRIAM, MetaShark, and EFICAz. 

## 2. Methods

### 2.1. Collection of Training Sequences

#### 2.1.1. Training Sequences from Enzyme/Swiss-Prot

**Figure 1:**
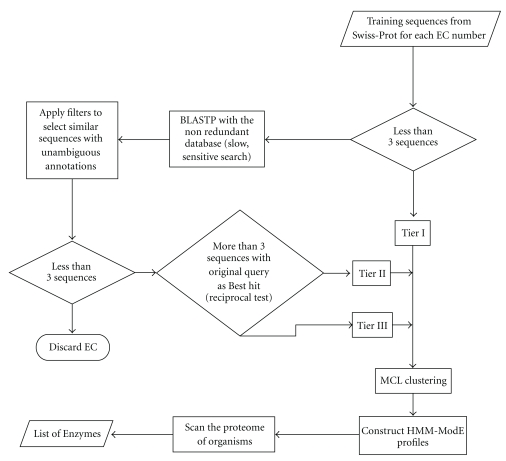
Flow diagram of the ModEnzA protocol.

The expasy enzyme database (release of 19 Jan 2010) had a total of 4905 unique EC number entries out of which 2507 were associated with a total of 180315 sequences. These 2507 entries were divided into 2 groups, those having 3 or more sequences and those having just 1 or 2 sequences. The 1910 EC numbers which had 3 or more sequences in Swiss-Prot were designated as Tier I (Figure 1) while the sequences in the latter group were used as queries to mine similar sequences from the nonredundant protein database (NR) database as follows.

#### 2.1.2. Mining of the Nonredundant Protein Database

There were 597 EC numbers that had just 1 or 2 sequences associated with them in the Swiss-Prot database. These were used as BLASTp queries to mine similar sequences from the NR database at NCBI (http://www.ncbi.nlm.nih.gov/sites/entrez?db=Protein). We used an *E*-value cut-off of 10^−35^, a percent identity range of 50 < ×<99 and a query coverage of 80% as the primary filters. If a sequence appeared as BLAST hit in more than one EC numbers, it was removed from all the EC number files. We removed sequences with ambiguous annotations such as “hypothetical”, “unnamed”, “unknown”, “unclassified”, and “unidentified”. While we did not follow a systematic testing approach to fix these parameters, we nevertheless tried various combinations of the parameters (*e*-values of 10^−30^ and 10^−35^, percent identity ranges 50–99, 75–99, 75–95, etc). The final set of parameters was decided upon after visual inspection of the annotations of the gathered sequences looking for the least amount sequences with ambiguous sequence descriptions and the most number of EC groups with more than 3 sequences.

After the filtering step, we were left with 450 EC number groups with 3 or more sequences. To these we further applied a reciprocal BLAST best hit criterion, wherein we discarded those NR sequences which did not have the original query swissprot sequence as their top hit. We obtained 364 EC groups having 3 or more sequences which fit this criterion. These were designated as Tier II profiles (Figure 1). The remaining 86 EC groups which also had 3 or more sequences but did not have sufficient reciprocal best hits were designated as Tier III (Figure 1).

### 2.2. Clustering Sequences Using the Markov Clustering Algorithm (MCL) [37]

The sequences belonging to each EC number in Tier I were clustered into subgroups with MCL using pairwise BLAST scores as input. This resulted in 2313 distinct subgroups having 3 or more sequences. These were considered as separate profiles called as Tier I profiles, for example the 7 subgroups of EC number 3.1.1.4 were designated as 3.1.1.4_1 through 3.1.1.4_7 and converted into separate HMMs. There were 117 EC numbers which had subgroups containing just 1 or 2 sequences. These subgroups were discarded after manual inspection. Similarly, the sequences in the other tiers were also clustered using MCL. We obtained 370 subgroups with more than three sequences in Tier II and 86 subgroups in Tier III making a total of 2769 distinct ModEnzA profiles.

### 2.3. HMM-ModE Profiles

The subgroups for each EC number were used to construct HMM profiles and HMM-ModE profiles as previously described [35]. Briefly, HMMs are generated for each subgroup using *hmmbuild* from the HMMER package [21] after aligning the sequences using MUSCLE [38]. The discrimination threshold is optimised by performing an *n*-fold cross-validation routine, partitioning the training sequences into *n* train and test sets such that each sequence is part of at least one test set. For each test set *t*, a profile HMM created from the remaining (*n* − 1) sets is used to score the sequences to get a True Positive (TP) score distribution. False positives (FP) are identified from the Swiss-Prot sequences (i.e those sequences that perform an enzymatic function which is different from that of the EC number for which the HMM was generated) using *hmmsearch* from HMMER. The FPs are also partitioned into *n* sets such that each FP sequence is part of at least one set. The profile HMM created for each (*n* − 1) subset is also used to score the corresponding FP set to get an FP score distribution. The sensitivity, specificity, and Matthews correlation coefficient (MCC) [39] distributions for each of *n* sets is calculated using these TP and FP score distributions. The optimal discrimination threshold is identified as the mid-point of the MCC distribution averaged over the *n* sets. 

The number of cross-validation sets *n* is defined as follows:

(1) $$\begin{array}{c} n=10,\;\forall \;\;\text{subgroups with sequences}\geq 10,\\ \begin{array}{cc} n=\left\{\begin{array}{c} \text{Number of FP if}\;\;\text{FP}<TP\\ \text{Number of TP if}\;\;\text{FP}>TP \end{array}\right\} & \\ \forall \;\;\text{subgroups with sequences}<10. & \end{array} \end{array}$$

If more than 3 false positives are identified, then these are again aligned and converted into HMMs and used to modify the true positive profile HMM as described earlier [35]. In case of a large number of false positives from the preclassified training set, to avoid issues of multiple alignments of very large datasets, we restrict the number of false positives to 200. The false positives are first clustered using MCL, and then sequences are randomly selected from the clusters proportionate to the size of the clusters such that the final number is 200. An optimized threshold is then calculated as above. In case no false positives are selected by the original HMM, it is used with default parameters. 

All methods described above were automated using scripts written in-house, to form the workflow described in Figure 1, and are available from the authors upon request.

### 2.4. Benchmark Datasets and Enzyme Identification Programs

For the bacterial genomes (*E. coli*, *B. aphidicola*, and *M. pneumoniae*) we used the corresponding HAMAP [40] annotations as the benchmark for comparing the various enzyme identification methods. The genome sequences for these and the corresponding annotations were downloaded from http://expasy.org/sprot/hamap/bacteria.html. We used *hmmsearch* to score the protein sequences for a given genome with the ModEnzA profiles generated above. 

The sequence IDs identified by each of the methods which had a 4-digit EC number annotation in HAMAP were considered as true positives (TP). False negatives (FN) were those predicted sequence IDs which had EC annotations in HAMAP but were not selected by a method whereas false positives (FP) were predicted sequences which actually did not have an EC number annotation in HAMAP. Similarly, for assigning EC numbers, true positives were those predicted EC numbers which had corresponding sequences in the HAMAP genome annotations, false positives, those that did not and false negatives were EC numbers which were present in HAMAP but not predicted by a method. For the *P. falciparum* genome, we used EC annotations in PlasmoDB as the benchmark. The sequence annotation and EC number information for *P. falciparum* can be obtained from http://plasmodb.org/plasmo/. True positives, false positives, and false negatives were defined as earlier. For the ROC curves for *P. falciparum* we used the KEGG annotations as the benchmark.

The PRIAM program [22] and the corresponding profiles were downloaded from http://priam.prabi.fr/REL_JUL06/index_jul06.html and used with an RPS-BLAST *e*-value cutoff of 10^−30^on the protein sequences of the genomes. The MetaShark package [24] was obtained from http://bmbpcu36.leeds.ac.uk/shark/ This was run using an *e*-value cutoff of 10^−30^ on the genomic DNA sequences of the organisms (including plasmids in case of *B. aphidicola*) obtained from NCBI ftp://ftp.ncbi.nih.gov/ The EFICAz enzyme annotations [25] for various organisms were obtained from http://cssb2.biology.gatech.edu/EFICAz/. 

The percentage sensitivity and specificity of each method was calculated as

(2) $$\begin{array}{c} \text{sensitivity}=\left(\frac{\text{TP}}{\left(\text{TP}+\text{FN}\right)}\right)*100,\\ \text{specificity}=\left(\frac{\text{TP}}{\left(\text{TP}+\text{FP}\right)}\right)*100. \end{array}$$

### 2.5. Receiver-Operator Characteristic Curves

**Figure 2:**
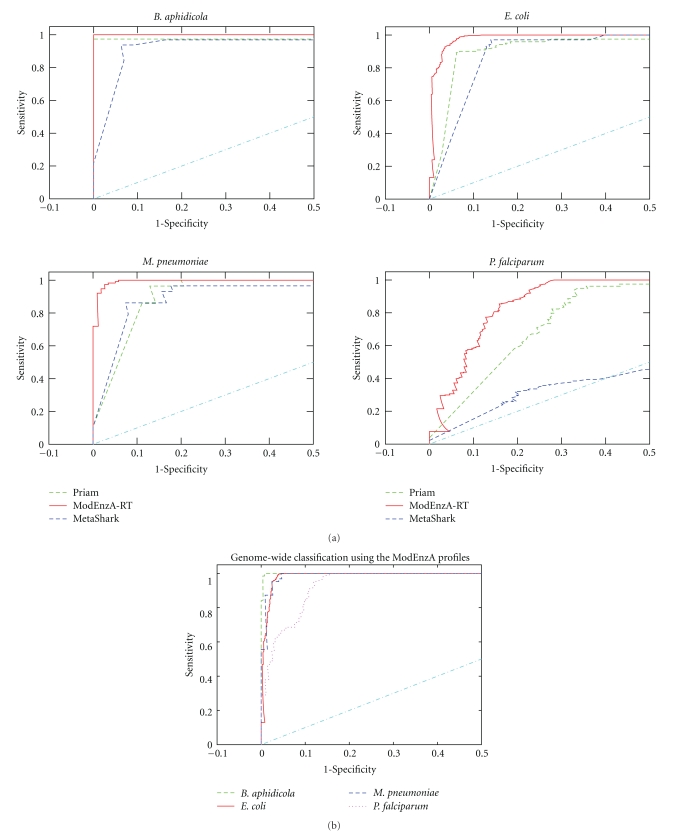
ROC curves for genome-wide enzyme identification using the ModEnzA profiles. The classification of the complete genomes of the four organisms is shown in (a). A fraction of the EC number profiles (284 out of 2075 Tier I ModEnzA profiles) were retrained with an older version of the ENZYME database and compared to PRIAM and MetaShark (b). ModEnzA-RT-Retrained ModEnzA profiles.

For comparison of ModEnzA with PRIAM and MetaShark the ModEnzA profiles for all EC numbers were rebuilt using the July 2006 version of ENZYME database which is used by the current versions of both PRIAM and MetaShark. The ROC curves (1-specificity versus sensitivity) were plotted for each of the three methods (PRIAM, MetaShark, and ModEnzA) on the four genomes by comparing against the benchmark sets mentioned above. The three programs were run on the genomes using an *E*-value threshold (RPS-BLAST, PSI-BLAST, and *hmmsearch E*-values for PRIAM, MetaShark, and ModEnzA, resp.) of 10. For PRIAM and MetaShark, the resulting *E*-values were binned and a threshold sweep was used to calculate the sensitivity and specificity at each *E*-value threshold. For ModEnzA, similar calculations were performed using a sweep through the *hmmsearch* scores (Figure 2(a)). The MetaShark output consists only of EC numbers, hence its sensitivity and specificity values were calculated only on the basis of EC number comparisons, whereas for PRIAM and ModEnzA, both the correct EC numbers predicted as well as the correct sequences assigned were used.

## 3. Results and Discussion

### 3.1. Enriching the Training Dataset

One of the crippling issues in biological data modeling is the incomplete nature of the training data. We have used both Swiss-Prot [41] and NR sequences for generating profiles specific for EC numbers. There were 1910 EC numbers which had 3 or more Swiss-Prot sequences associated with them. These form our Tier I profiles, which can be used to annotate sequences with a very high degree of confidence, since they were generated from curated training sequences. We considered three sequences as the minimum number required to generate a multiple alignment and consequently for constructing HMMs. Even though these profiles with 3 sequences (and in general all profiles with very few training sequences) are not expected to be representative of the protein function, they are, nevertheless, included in the ModEnzA protocol as place holders which would become more accurate as they are populated with more sequences in future.

The training set for EC numbers which had less than 3 Swiss-Prot sequences was enriched by mining sequences from the NR database using BLASTp with parameters suitably modified for increased sensitivity (namely, BLOSUM62 substitution matrix and a lower than default value for “*f*”, the hit extension parameter). The hits were screened with a stringent *E*-value cut-off of 10^−35^. Hits having more than 99% identity or those having less than 50% identity with the query sequence were left out to remove identical sequences and potential false positives while ensuring variability in the protein family. This resulted in 450 EC groups (which had more than three sequences) out of which in 364 EC groups (Tier II), all the mined sequences picked up the original swissprot query sequence as the reciprocal best BLAST hit. The remaining 86 were put in a separate group designated as Tier III. By enriching the training data set, we have populated an additional 450 enzyme functions with high-quality sequences, providing increasing coverage over the set of enzyme functions. This is still not comprehensive—the coverage that our profiles provide, including Tier II and III, is still only 48% of the total number of enzyme functions known. However, the same method developed for known functions may be applied to new functions as they are mapped with sequences in future versions, of which this enrichment exercise serves as an example. The coverage within an organism is expected to be much higher, as most common functions are mapped with known sequences.

### 3.2. Clustering Training Sequences with Markov Clustering Algorithm (MCL)

Sequences belonging to the same EC group and thus performing the same enzymatic function, might have complex relationships amongst them in terms of sequence similarity due to the presence of (1) heteromeric multiple subunits of the enzymes, (2) nonorthologous or unrelated sequences performing the same function, or (3) sequences that can perform more than one enzymatic function [22]. Clustering the sequences for a particular EC number based on a similarity score is therefore an important requirement to separate these multiple subunits or nonorthologous sequences. 

We used MCL to cluster the sequences of an EC group into subgroups after removing sequences which had “fragment” as part of the annotation. To validate our use of automated clustering, we compared our MCL clusters for some of the cases mentioned above with available structural information from PDB [42]. For example, MCL clusters the swissprot sequences for the DNA-directed RNA polymerase (EC 2.7.7.6) into 14 subgroups (PRIAM has 51 separate profiles for EC 2.7.7.6). We tested the ModEnzA profiles for EC 2.7.7.6 on a set of sequences of the structural subunits for the RNA polmerase downloaded from PDB. The yeast polymerase (PDB ID: 3H0G) has 12 subunits while the prokaryotic polymerase from *E. coli* (PDB ID: 3LU0) has 5 subunits (excluding the sigma subunit). In each case, the sequences corresponding to different subunits were picked up by a different ModEnzA subgroup profile (Supplementary Table ST2 available online at doi:10.1155/2011/743782). We also manually inspected the “singlet” sequences, that is the sequences which the MCL clustering procedure cannot assign to any cluster bigger than size 3 and thus have to be discarded while making the profiles. We obtained 2313 distinct MCL subgroups with more than 3 sequences in Tier I. There were 117 EC numbers (with a total of 215 “singlet” sequences) with subgroups having less than 3 sequences. These were subjected to a BLASTp analysis against the NR database and the results were manually inspected to ascertain the functional neighbours (top BLAST hits) of these sequences. Out of the 215 sequences that were so tested, 23% (55) matched with sequences which had a different functional annotation than the original EC group suggesting that these might have had possible errors in annotation. Some of the sequences (14 or 6%) were shorter than most of the other sequences in that EC group (probably fragments or truncated proteins) whereas a fraction (3%) were longer than the rest. Another 15 sequences (6%) were not clustered because they belonged to a different sub-unit of the enzyme, indicating the ability of MCL to clearly distinguish between heteromeric subunits of enzymes. Whereas 23% sequences did not show any significant hits in the BLAST search, a sizable fraction (30%) were such that they had the correct annotation but not a sufficient number of neighbours to form a separate cluster. These were not included in the profile construction; however, they could be used for mining similar sequences from the NR database if they match sequences with the same function as the EC group. As mentioned above, all of the 215 sequences were not included in the profile generation step. This exercise demonstrates the efficacy of using an MCL-based clustering approach in separating enzyme subgroups.

The PRIAM method identifies the longest homologous subsequences shared within the set of sequences associated with EC number (EC group) as a single module or domain. It initially takes the shortest sequence in the group as a module and then proceeds to identify similar subsequences within a given EC group using PSI-BLAST. The matching subsequences are removed from the corresponding sequences and the shortest sequence identified to start the next iteration [22]. Complete linkage clustering using a 30% sequence identity cutoff has also been employed to create subgroups as in the EFICAz protocol [25, 26]. The most populated and most divergent subgroup is then converted into a profile HMM which is used to add sequences with *E*-value <0.01 which have at least one conserved potential active site residue. These subgroups are termed as CHIEFc (conservation-controlled HMM iterative procedure for enzyme family classification) families [25]. The Markov clustering algorithm (MCL) provides for an accurate, unsupervised, and fully automated protocol for clustering the sequences of a given EC number. MCL avoids potential problems associated with module architecture-based clustering as well as the pairwise protocols of dealing with similarity relationships which have been used earlier [43]. It also circumvents the requirement of using arbitrary sequence identity cutoffs as described above or manual assignment. Instead, the graph-theoretic representation of the similarity scores allows for the detection of global patterns of sequence similarity in a single step [37].

### 3.3. Validation and Comparison of ModEnzA with Existing Methods

The training sequences for each EC number were used to generate HMM profiles using the HMM-ModE protocol as described in Methods. The average sensitivity and specificity distributions of the *n* sets in the *n*-fold cross validation exercise provide us with a confidence measure with which to use the profiles. If the original profile HMM selects false positives from within the negative training sequences, the information from these is used to modify the emission probabilities of the HMM so that it becomes more specific. 

The ModEnzA profiles were used to scan the complete genomes of organisms to assess the performance of our method. Following the example of [22, 24], we chose three bacterial genomes (*E. coli*, *B. aphidicola*, and *M. pneumoniae*) which have been extensively annotated using both manual and automatic methods as part of the high-quality automated and manual annotation of microbial proteomes project [40] and one eukaryotic genome (*P. falciparum*), for which detailed descriptions of function are available from the PlasmoDB resource [44]. The enzymatic function annotations from these sources were used as benchmarks for comparing the sensitivity and specificity of ModEnzA with other enzyme identification methods PRIAM, MetaSHARK, and EFICAz (see Section 2). 

Identification of functional residues or residues that are responsible for imparting functional specificity is a critical step toward constructing function specific profiles. Information theoretic measures such as positional entropy [45] and mutual information [46] have earlier been used for this purpose. EFICAz employs an evolutionary footprinting approach which scores each position in an alignment of a family of sequences by a combination of entropy based conservation scores in (1) the “homofunctional” alignment, that is, consisting of members of the same family and (2) a “heterofunctional” alignment which consists of similar sequences (which might have different functions), mined from a nonredundant database using the homofunctional profile HMM. The alignment positions are then ranked using a Z-score of the conservation degree to identify functionally discriminating residues (FDRs). HMM-ModE, the protocol upon which ModEnzA is based, uses a similar concept of negative training sequences, these are sequences belonging to other functional families which are scored positively by the HMM for a particular family. We use a position-dependent null model which contains conservation information from these negative training sequences. We calculate the relative entropy between the distributions of amino acids in the alignments of the positive and negative training sequences and select residues where this score is higher than the relative entropy between the positive sequences and the null set (i.e., the null probabilities as calculated from the swissprot sequences). The probabilities of the amino acids in the negative set are used to modify the emission probabilities of these selected residues to generate a function specific profile HMM. This avoids the problem of loss of information associated with using a Z-score cutoff [35]. The discrimination potential of a profile is a function of the unique nature of the family at the level of both its fold and specificity determining positions. As a consequence, the discriminating potential of a profile HMM at a functional level would be expected to be dependent on the frequency of occurrence of the parent fold among proteins, that is, an activity that arises in a commonly occurring fold will be more likely to have false positives from the complete training set than an activity that arises in a fold that is not so wide-spread. As such, there is no discernible relationship between the cluster size (no. of training sequences available for a subgroup) and the discrimination potential of the corresponding profile (Supplementary Figure SF2 available online at doi:10.1155/2011/743782). 

EFICAz uses a family specific Sequence Identity threshold (SIT), which is set after making all pairwise sequence comparisons within the family, for predicting enzyme function [25]. Both PRIAM and MetaShark have options for varying the *e*-value threshold. An *E*-value cutoff of 10^−10^ in PRIAM results in a high sensitivity (92.61%) but a specificity of only 82.25% for identifying EC numbers from *E. coli*. On the other hand, using a stringent *E*-value cutoff of 10^−30^ increases specificity (90.42%) with a drop in sensitivity (90.80%) (data not shown). This is a drawback of using arbitrary thresholds for discrimination. ModEnzA uses cross-validation to ensure an optimal threshold for each separate profile [32] thereby eliminating the use of arbitrary thresholds. 

The EFICAz webserver at http://cssb2.biology.gatech.edu/cgi-bin/eficaz_browse.cgi provides 4 digit EC number predictions for a number of genomes. These predictions were directly compared with ModEnzA. The sensitivity and specificity were calculated with respect to the EC numbers that were correctly assigned to a genome as well as the sequences associated with an enzymatic activity which were identified by the methods. The comparative performance of EFICAz and ModEnzA for identifying EC numbers and enzymatic sequences from the four genomes is shown in Table 1. The sensitivity and specificity values of the ModEnzA profiles are higher compared to EFICAz both in assigning EC numbers as well as identifying enzymatic sequences. As expected, the sensitivity is improved by the inclusion of the Tier II and Tier III profiles (the sensitivity in assigning EC-associated sequences increasing up to 97% for *B. aphidicola*, e.g.) but there is a slight drop in the specificity because the training sequences were mined from the NR database and hence may have errors of annotation. The Tier I ModEnzA profiles by themselves show a higher specificity than EFICAz while selecting enzymatic sequences for each genome tested. The augmentation by Tier II and III profiles still maintains an on par specificity while increasing the sensitivity to a value greater than that of EFICAz for identifying both EC numbers and sequences. Given that the genome databases mentioned above also have an automated annotation component to them, it must be noted that choosing a different set of annotations as the benchmark (e.g KEGG) does not significantly alter the conclusions of the comparisons (supplementary Table ST3 available online at doi:10.1155/2011/743782).

The PRIAM and MetaShark methods use the July 2006 version of the ENZYME database as the training set. To ensure a fair comparison we rebuilt all the ModEnzA profiles using the July 2006 ENZYME version. As demonstrated in the next section, the KEGG database has more EC-sequence associations for *P. falciparum *than PlasmoDB, and for this reason we replaced PlasmoDB with KEGG as the benchmark for the comparison of ModEnzA with PRIAM and MetaShark. Receiver-operator characteristic (ROC) curves serve as an indicator for the discriminating potential of a classifier method. The ROC curves for the retrained ModEnzA (ModEnzA-RT) profiles as well as PRIAM and MetaShark are shown in Figure 2(a). Since EFICAz uses 4 different methods, each with its own set of parameters, we could not include EFICAz in this analysis. The area under the ROC curve for the ModEnzA profiles is better than both PRIAM and MetaShark for all the four genomes. MetaShark predictions on *P. falciparum* suffer from very low specificity (Figure 2(a), bottom left panel) probably because it is difficult to map the profile HMMs back onto its DNA sequence which is atypically AT rich and contains lots of repetitive sequences [47]. The ROC curves for ModEnzA scans on the entire genomes of the four organisms (Figure 2(b)) show the high discrimination potential of the ModEnzA profiles.

### 3.4. Enzyme Identification from the *P. falciparum* Genome

Eukaryotic species account for only about 16% of the most represented species (31% of all sequences) in terms of number of sequence entries in Swiss-Prot (http://ca.expasy.org/sprot/relnotes/relstat.html). The *P. falciparum* genome is also not included in the HAMAP project which deals extensively with bacterial, archaeal, and plastid encoded proteins. Hence, this could be considered as an ideal case to compare the various enzyme identification methods. We chose the PlasmoDB enzyme annotations as a benchmark to decide on the true positive, false positive, and false negative predictions. The sensitivity and specificity calculated in terms of these numbers for ModEnzA and EFICAz is shown in Table 1. The relative scarcity of training sequences from the eukaryotic domain is reflected in the low sensitivities of the methods. Again the specificity of the ModEnzA profiles is higher than EFICAz.

ModEnzA assigned 22 EC numbers which were not annotated in PlasmoDB (False Positives). We checked for the corresponding functions of these EC numbers in two other databases, namely KEGG (because it is often used as a reference knowledge base) [12] and PlasmoCyc [4] (which also contains a comprehensive annotation of the *P. falciparum* genome). We found that 13 of the 22 false positive EC numbers have been annotated as belonging to *P. falciparum* by either of these databases (Table 2).

We were interested in the ability of Tier II and Tier III profiles to annotate novel sequences, especially as they were created from sequence similarity and not expert-curated data sets. It was also interesting to address the fact that in the absence of a profile, ModEnzA could be used to pick a related function. The Tier II ModEnzA profiles selected 14 sequences and 7 EC numbers, respectively, from the *P. falciparum* genome (Table 3). The cysteine protease falcipain sequences (gene IDs PF11_0161, PF11_0162, and PF11_0165), for instance, do not currently have an EC number associated with them. So in absence of a corresponding EC profile, ModEnzA annotates it with the nearest cysteine endopetidase bromelain (EC 3.4.22.32) but it is gratifying to note that the annotation is correct upto the general level of the first three E.C. digits. Three of the 7 EC numbers predicted by ModEnzA exactly match the corresponding PlasmoDB assignments while 3 others have the same first three digits. Only one EC assignment (EC 3.4.23.2) has more than 1 digit mismatch with the EC annotated in PlasmoDB. Of the 14 sequences annotated, the EC assignments for 8 share the first three digits with the corresponding annotations in PlasmoDB (Table 3). The Tier II and Tier III profiles can annotate sequences up to the first three EC digits with sufficient accuracy. However, as has been mentioned earlier, these should be used with caution, because the training sequences used for these profiles may be prone to annotation errors.

As the results show, there is a discrepancy between the annotations/predictions of different databases. The ModEnzA protocol assumes importance because it is a rapid tool that provides a high degree of confidence in assigning EC numbers to a genome. A typical *hmmsearch* with ModEnzA Tier I profiles on the *E. coli* genome (4407 proteins) takes ~7.8 Hrs on a laptop having a 2.4 GHz core2duo Intel processor and 3 Gb RAM. The same search on a workstation with a 2.50 GHz Intel xeon quadcore processor with 8 GB RAM takes ~2.2 Hrs. Since the *hmmsearch* and *hmmscan* program is inherently capable of multithreading, ModEnzA can be expected to be even faster on machines with more processors. For example, we were able to annotate the enzymes in a metagenomic sample with 203240 translated protein fragments with ModEnzA in just around 1.3 hrs by splitting the target sequences into 10 parts and using 10 nodes (each with a dual core processor) to run the *hmmscan* program on a high-performance computing cluster (unpublished results). The *n*-fold cross validation routine built into ModEnzA on the training sequences ensures an optimal threshold which can separate the true positives from the False positives for any given EC number. The modification of emission probabilities of the True positive profiles by using information from the false positive alignment further increases the specificity. We have decided to make this data available for use by the scientific community using HMMER2, even though HMMER3 has since been released [21]. HMMER3 has only local-local alignments, and our method is based on predicting the fold (domain) and hence is implicitly based on global or “Glocal” (align a complete model to a subsequence of the target) alignments. ModEnzA is based on scripts that modify the emission probabilities in the model, and it is unsure if the formats for HMMER3 are stable enough to extract probabilities from the model. The model has changed between the two versions, and it has been advised that profiles built on one should not be used with the other, as parameters are differently optimised. When more alignment modes are available and the format is more stable, newer versions of ModEnzA would migrate to HMMER3 to take advantage of the increased sensitivities and speed.

## 4. Conclusion

We present a method for enzyme annotation by enriching existing curated databases and using profile hidden Markov models optimised for specificity using negative training sequences. The protocol shows improved sensitivity and specificity compared to other existing methods for enzyme identification and can be used to accurately map the metabolome of an organism.

## Supplementary Material

Supplementary data includes 3 tables and 2 figures related to the paper.
Table ST1 gives the EC numbers and functional description of some enzymes belonging to the Alpha/Beta Hydrolase fold.
Table ST2 provides the correspondence between the subclusters or subgroups of the ModEnzA profile for EC 2.7.7.6 (DNA-directed RNA polymerase) and its structural subunits.
Table ST3 shows the comparison between ModEnzA and EFICAz for enzyme identification from 4 completely sequenced genomes using the KEGG annotations for these genomes as the benchmark.
Figure SF1 shows the structural similarity in the folds of six enzymes belonging to the Alpha/Beta hydrolase fold. The inset shows the proportion of False Positives selected from the EC groups by the HMM profile of EC 3.1.1.8.
Figure SF2 is the plot of the cluster-size of the training sequences and their discriminative potential as indicated by their Average Matthews Correlation Co-efficient (MCC) values.

## Figures and Tables

**Table 1:** Genome-wide enzyme identification for three bacterial genomes (*E. coli*, *B. aphidicola*, and *M. pneumoniae*) and one eukaryotic genome (*P. falciparum*) by ModEnzA and EFICAz.

Methods		EFICAz	ModEnzA(Tier I)	ModEnzA(Tier I+II)
Annotation benchmark	HAMAP			

*E. coli*				
Sequences	1012	859 (1051)	902 (1021)	930 (1082)
Sensitivity		84.88	89.13	91.89
Specificity		81.73	88.34	85.95
EC numbers	755	653 (728)	663 (697)	699 (775)
Sensitivity		86.49	87.81	92.58
Specificity		89.69	95.12	90.19
*B. aphidicola*				
Sequences	273	257 (273)	264 (271)	265 (273)
Sensitivity		94.13	96.7	97.07
Specificity		94.13	97.41	97.07
EC numbers	245	226 (238)	225 (229)	225 (233)
Sensitivity		92.24	91.83	91.83
Specificity		94.95	98.25	96.56
*M. pneumoniae*				
Sequences	147	119 (149)	126 (139)	126 (139)
Sensitivity		80.95	85.71	85.71
Specificity		79.86	90.64	90.64
EC numbers	127	101 (122)	115 (122)	115 (122)
Sensitivity		79.52	90.55	90.55
Specificity		82.78	94.26	94.26

Annotation benchmark	PlasmoDB			

*P. falciparum*				
Sequences	771	341 (480)	350 (415)	358 (431)
Sensitivity		44.22	45.39	46.43
Specificity		71.04	84.33	83.06
EC numbers	410	217 (247)	212 (234)	215 (242)
Sensitivity		52.92	51.7	52.43
Specificity		87.85	90.59	88.84

Numbers within parentheses indicate the total number of sequences or EC numbers identified by each method.

**Table 2:** Conflicting annotations for the 22 EC numbers predicted as belonging to the *P. falciparum* genome by ModEnzA but not annotated in PlasmoDB.

EC No.	Sequence	KEGG	PlasmoCyc
1.1.5.3	PFC0275w	FAD-dependent glycerol-3-phosphate dehydrogenase, putative	FAD-dependent glycerol-3-phosphate dehydrogenase, putative
1.17.7.1	PF10_0221	(E)-4-Hydroxy-3-methylbut-2-enyl-diphosphate synthase	Methylerythritol phosphate pathway
1.3.1.8	PF11_0370	—	—
1.3.5.2	PFF0160c	—	Uridine-5′-phosphate biosynthesis
2.1.1.48	PF14_0156	—	—
2.3.1.180	PFB0505c	3-Oxoacyl-(acyl carrier protein) synthase III, putative	Fatty acid biosynthesis initiation I
2.3.1.181	MAL8P1.37	Lipoyl(octanoyl) transferase	—
2.4.1.141	MAL8P1.133	Beta-1,4-N-acetylglucosaminyltransferase	Dolichyl-diphosphooligosaccharide biosynthesis
2.7.12.1	PF14_0431	dual-specificity kinase	—
2.7.1.90	PFI0755c	6-phosphofructokinase	ATP-dependent phosphofructokinase, putative
2.7.7.64	PFE0875c	—	—
2.8.1.8	MAL13P1.220	Lipoic acid synthetase	—
3.1.13.4	MAL8P1.104, PFE0980c	—	—
3.1.21.2	PF13_0176	—	—
3.4.21.10	PFE0340c, PF11_0149, MAL8P1.16, PF14_0110	—	—
3.5.1.88	PFI0380c	—	—
3.6.1.1	PF14_0541, PFL1700c, PFC0710w-a, PFC0710w-b	Inorganic pyrophosphatase	Inorganic pyrophosphatase, putative, V-type H(+)-translocating pyrophosphatase, putative
3.6.1.7	PF11_0121	—	—
3.6.3.44	PFE1150w	—	ABC transporter, putative
3.6.4.3	PF14_0548	—	—
3.6.4.6	PFC0140c	Vesicle-fusing ATPase	—
3.6.5.5	PF10_0368	Dynamin GTPase	—

“—”–Annotation not present in either PlasmoCyc or KEGG.

**Table 3:** Three-digit annotations for the sequences selected from *P. falciparum* by Tier II and Tier III profiles.

Gene	PlasmoDB product description*	PlasmoDB EC*	ModEnzA EC	EC description^#^
PF07_0059	4-nitrophenylphosphatase, putative	3.1.3.-(Phosphoric monoester hydrolases.); 3.1.3.41 (4-nitrophenylphosphatase)	T2-3.1.3.41	4-nitrophenylphosphatase
PF08_0108	Pepsinogen, putative	3.4.23.1	T2-3.4.23.2	Pepsin B
PF10_0329	Aspartyl protease, putative; Plasmepsin VII	None	T2-3.4.23.2	Pepsin B
PF11_0161	Falcipain-2 precursor, putative	3.4.22.-	T3-3.4.22.32	Stem bromelain
PF11_0162	Falcipain-3	3.4.22.-	T3-3.4.22.32	Stem bromelain
PF11_0165	Falcipain 2 precursor	3.4.22.-	T3-3.4.22.32	Stem bromelain
PF11_0295	Farnesyl pyrophosphate synthase, putative	2.5.1.10 Geranyltranstransferase; 2.5.1.1 Dimethylallyltranstransferase	T2-2.5.1.67	Chrysanthemyl diphosphate synthase
PF14_0075	Plasmepsin, putative	3.4.23.38 (Plasmepsin I)	T2-3.4.23.39	Plasmepsin II
PF14_0076	Plasmepsin 1 precursor	3.4.23.38 (Plasmepsin I)	T2-3.4.23.39	Plasmepsin II
PF14_0077	Plasmepsin 2	3.4.23.39 (Plasmepsin II)	T2-3.4.23.39	Plasmepsin II
PF14_0078	HAP protein; Plasmepsin III	3.4.23.-Aspartic endopeptidases	T2-3.4.23.39	Plasmepsin II
PF14_0281	Aspartyl protease, putative	None	T2-3.4.23.2	Pepsin B
PF14_0334	NAD(P)H-dependent glutamate synthase, putative	1.4.7.1 Glutamate synthase (ferredoxin);1.4.1.14 -Glutamate synthase (NADH)	T2-1.4.1.14	Glutamate synthase
PF14_0553	Cysteine proteinase falcipain-1	None	T3-3.4.22.32	Stem bromelain
PF14_0625	Hypothetical protein	3.4.2.3; Transferred entry: 3.4.17.4	T2-3.4.23.2	Pepsin B
PFC0495w	Aspartyl protease, putative	3.4.23.- Aspartic endopeptidases	T2-3.4.23.2	Pepsin B
PFF0530w	Transketolase, putative	2.2.1.1 Transketolase	T2-2.2.1.3	Formaldehyde transketolase
PFI1125c	3-oxoacyl-(acyl-carrier protein) reductase, putative	1.1.1.100 (3-oxoacyl-[acyl-carrier-protein] reductase); 2.3.1.85 (Fatty-acid synthase)	T2-1.1.1.140	Sorbitol-6-phosphate 2-dehydrogenase

*Gene product descriptions and EC annotations obtained from PlasmoDB. ^#^IUBMB EC description.

## References

[1] MacLean D, Jones JDG, Studholme DJ (2009). Application of ’next-generation’ sequencing technologies to microbial genetics. *Nature Reviews Microbiology*.

[2] Galperin MY, Koonin EV (1999). Searching for drug targets in microbial genomes. *Current Opinion in Biotechnology*.

[3] Hopkins AL, Groom CR (2002). The druggable genome. *Nature Reviews Drug Discovery*.

[4] Russ AP, Lampel S (2005). The druggable genome: an update. *Drug Discovery Today*.

[5] Yeh I, Hanekamp T, Tsoka S, Karp PD, Altman RB (2004). Computational analysis of *Plasmodium falciparum* metabolism: organizing genomic information to facilitate drug discovery. *Genome Research*.

[6] Price HP, Menon MR, Panethymitaki C, Goulding D, McKean PG, Smith DF (2003). Myristoyl-CoA: protein N-myristoyltransferase, an essential enzyme and potential drug target in kinetoplastid parasites. *Journal of Biological Chemistry*.

[7] Upcroft P, Upcroft JA (2001). Drug targets and mechanisms of resistance in the anaerobic protozoa. *Clinical Microbiology Reviews*.

[8] Hasan S, Daugelat S, Rao PSS, Schreiber M (2006). Prioritizing genomic drug targets in pathogens: application to Mycobacterium tuberculosis. *PLoS Computational Biology*.

[9] Anishetty S, Pulimi M, Pennathur G (2005). Potential drug targets in Mycobacterium tuberculosis through metabolic pathway analysis. *Computational Biology and Chemistry*.

[10] Rodaki A, Young T, Brown AJP (2006). Effects of depleting the essential central metabolic enzyme fructose-1,6-bisphosphate aldolase on the growth and viability of *Candida albicans*: implications for antifungal drug target discovery. *Eukaryotic Cell*.

[11] Morgunova E, Saller S, Haase I (2007). Lumazine synthase from *Candida albicans* as an anti-fungal target enzyme: structural and biochemical basis for drug design. *Journal of Biological Chemistry*.

[12] Xu D, Jiang B, Ketela T (2007). Genome-wide fitness test and mechanism-of-action studies of inhibitory compounds in *Candida albicans*.. *PLoS Pathogens*.

[13] Rusch DB, Halpern AL, Sutton G (2007). The *Sorcerer II* Global Ocean Sampling expedition: northwest Atlantic through eastern tropical Pacific.. *PLoS Biology*.

[14] Gianoulis TA, Raes J, Patel PV (2009). Quantifying environmental adaptation of metabolic pathways in metagenomics. *Proceedings of the National Academy of Sciences of the United States of America*.

[15] Kanehisa M, Goto S, Hattori M (2006). From genomics to chemical genomics: new developments in KEGG. *Nucleic Acids Research*.

[16] Maltsev N, Glass E, Sulakhe D (2006). PUMA2—grid-based high-throughput analysis of genomes and metabolic pathways. *Nucleic Acids Research*.

[17] Caspi R, Foerster H, Fulcher CA (2008). The MetaCyc Database of metabolic pathways and enzymes and the BioCyc collection of pathway/genome databases. *Nucleic Acids Research*.

[18] Altschul SF, Gish W, Miller W, Myers EW, Lipman DJ (1990). Basic local alignment search tool. *Journal of Molecular Biology*.

[19] Pearson WR, Lipman DJ (1988). Improved tools for biological sequence comparison. *Proceedings of the National Academy of Sciences of the United States of America*.

[20] Altschul SF, Madden TL, Schäffer AA (1997). Gapped BLAST and PSI-BLAST: a new generation of protein database search programs. *Nucleic Acids Research*.

[21] Eddy SR HMMER: biological sequence analysis using profile hidden Markov models. http://hmmer.org/.

[22] Claudel-Renard C, Chevalet C, Faraut T, Kahn D (2003). Enzyme-specific profiles for metabolic pathway prediction: PRIAM. *Nucleic Acids Research*.

[23] Marchler-Bauer A, Panchenko AR, Shoemarker BA, Thiessen PA, Geer LY, Bryant SH (2002). CDD: a database of conserved domain alignments with links to domain three-dimensional structure. *Nucleic Acids Research*.

[24] Pinney JW, Shirley MW, McConkey GA, Westhead DR (2005). metaSHARK: software for automated metabolic network prediction from DNA sequence and its application to the genomes of *Plasmodium falciparum* and *Eimeria tenella*. *Nucleic Acids Research*.

[25] Tian W, Arakaki AK, Skolnick J (2004). EFICAz: a comprehensive approach for accurate genome-scale enzyme function inference. *Nucleic Acids Research*.

[26] Arakaki AK, Huang Y, Skolnick J (2009). EFICAz: enzyme function inference by a combined approach enhanced by machine learning. *BMC Bioinformatics*.

[27] Peregrin-Alvarez JM, Tsoka S, Ouzounis CA (2003). The phylogenetic extent of metabolic enzymes and pathways. *Genome Research*.

[28] Bairoch A (2000). The ENZYME database in 2000. *Nucleic Acids Research*.

[29] Green ML, Karp PD (2004). A Bayesian method for identifying missing enzymes in predicted metabolic pathway databases. *BMC Bioinformatics*.

[30] Kelley BP, Yuan B, Lewitter F, Sharan R, Stockwell BR, Ideker T (2004). PathBLAST: a tool for alignment of protein interaction networks. *Nucleic Acids Research*.

[31] Ye Y, Osterman A, Overbeek R, Godzik A (2005). Automatic detection of subsystem/pathway variants in genome analysis. *Bioinformatics*.

[32] Overbeek R, Fonstein M, D’Souza M, Push GD, Maltsev N (1999). The use of gene clusters to infer functional coupling. *Proceedings of the National Academy of Sciences of the United States of America*.

[33] Kharchenko P, Vitkup D, Church GM (2004). Filling gaps in a metabolic network using expression information. *Bioinformatics*.

[34] Kharchenko P, Chen L, Freund Y, Vitkup D, Church GM (2006). Identifying metabolic enzymes with multiple types of association evidence. *BMC Bioinformatics*.

[35] Srivastava PK, Desai DK, Nandi S, Lynn AM (2007). HMM-ModE—improved classification using profile hidden Markov models by optimising the discrimination threshold and modifying emission probabilities with negative training sequences. *BMC Bioinformatics*.

[36] Murzin AG, Brenner SE, Hubbard T, Chothia C (1995). SCOP: a structural classification of proteins database for the investigation of sequences and structures. *Journal of Molecular Biology*.

[37] van Dongen S (May 2000). *Graph clustering by flow simulation*.

[38] Edgar RC (2004). MUSCLE: multiple sequence alignment with high accuracy and high throughput. *Nucleic Acids Research*.

[39] Baldi P, Brunak S, Chauvin Y, Andersen CAF, Nielsen H (2000). Assessing the accuracy of prediction algorithms for classification: an overview. *Bioinformatics*.

[40] Gattiker A, Michoud K, Rivoire C (2003). Automated annotation of microbial proteomes in SWISS-PROT. *Computational Biology and Chemistry*.

[41] Apweiler R, Bairoch A, Wu CH (2004). UniProt: the universal protein knowledgebase. *Nucleic Acids Research*.

[42] Berman HM, Westbrook J, Feng Z (2000). The protein data bank. *Nucleic Acids Research*.

[43] Enright AJ, van Dongen S, Ouzounis CA (2002). An efficient algorithm for large-scale detection of protein families. *Nucleic Acids Research*.

[44] Bahl A, Brunk B, Crabtree J (2003). PlasmoDB: the Plasmodium genome resource. A database integrating experimental and computational data. *Nucleic Acids Research*.

[45] Hannenhalli SS, Russell RB (2000). Analysis and prediction of functional sub-types from protein sequence alignments. *Journal of Molecular Biology*.

[46] Li L, Shakhnovich EI, Mirny LA (2003). Amino acids determining enzyme-substrate specificity in prokaryotic and eukaryotic protein kinases. *Proceedings of the National Academy of Sciences of the United States of America*.

[47] Gardner MJ, Hall N, Fung E (2002). Genome sequence of the human malaria parasite *Plasmodium falciparum*. *Nature*.

