# Supplementary material for: ModEnzA: Accurate Identification of Metabolic Enzymes Using Function Specific Profile HMMs with Optimised Discrimination Threshold and Modified Emission Probabilities
Source: Adv Bioinformatics. 2011 Mar 29;2011:743782. doi: 10.1155/2011/743782 (PMC3085309; doi:10.1155/2011/743782)

Table ST1 EC numbers belonging to the Alpha/Beta Hydrolase fold

EC Numbers	Functional Description (from KEGG)
3.1.1.1	Carboxylesterase
3.1.1.3	Triacylglycerol lipase
3.1.1.7	Acetylcholinesterase
3.1.1.8	Cholinesterase
3.1.1.13	Sterol esterase
3.1.1.41	Cephalosporin-C deacetylase
3.1.1.43	Alpha-amino-acid esterase
3.1.1.72	Acetylxylan esterase
3.1.1.73	Feruloyl esterase
3.1.1.74	Cutinase

Table ST2 Structural subunits of DNA-directed RNA Polymerase (EC 2.7.7.6) selected by ModEnzA profiles for EC 2.7.7.6

PDB Sequence ID	ModEnzA subgroup profile	Score
3LU0:C|PDBID|CHAIN|SEQUENCE	2.7.7.6_2	3173.7
3LU0:E|PDBID|CHAIN|SEQUENCE	2.7.7.6_4	130.6
3LU0:A|PDBID|CHAIN|SEQUENCE	2.7.7.6_3	630.3
3LU0:B|PDBID|CHAIN|SEQUENCE	2.7.7.6_3	630.3
3LU0:D|PDBID|CHAIN|SEQUENCE	2.7.7.6_1	2033.3
3H0G:B|PDBID|CHAIN|SEQUENCE	2.7.7.6_2	909.1
3H0G:N|PDBID|CHAIN|SEQUENCE	2.7.7.6_2	909.1
3H0G:F|PDBID|CHAIN|SEQUENCE	2.7.7.6_4	16.1
3H0G:R|PDBID|CHAIN|SEQUENCE	2.7.7.6_4	16.1
3H0G:E|PDBID|CHAIN|SEQUENCE	2.7.7.6_6	98.6
3H0G:Q|PDBID|CHAIN|SEQUENCE	2.7.7.6_6	98.6
3H0G:J|PDBID|CHAIN|SEQUENCE	2.7.7.6_5	109.7
3H0G:V|PDBID|CHAIN|SEQUENCE	2.7.7.6_5	109.7
3H0G:L|PDBID|CHAIN|SEQUENCE	2.7.7.6_7	22.9
3H0G:X|PDBID|CHAIN|SEQUENCE	2.7.7.6_7	22.9

3H0G:A|PDBID|CHAIN|SEQUENCE	2.7.7.6_1	1165.1
3H0G:M|PDBID|CHAIN|SEQUENCE	2.7.7.6_1	1165.1
3H0G:G|PDBID|CHAIN|SEQUENCE	2.7.7.6_1	25.2
3H0G:S|PDBID|CHAIN|SEQUENCE	2.7.7.6_14	25.2

The structure **3LU0** has in total **5** chains. Out of these **4** are sequence-unique. Chain A and B are identical.
The structure **3H0G** has in total **24** chains. Out of these **12** are sequence-unique. The identical pairs are A-M, J-V, K-W, L-X, B-N, C-O, D-P, E-Q, F-R, G-S, H-T and I-U

Table ST3 Genome-wide enzyme identification for three bacterial genomes (*E. coli* , *B. aphidicola* and *M. pneumoniae*) and one eukaryotic genome (*P. falciparum*) by ModEnzA and EFICAz using KEGG annotations as benchmark

**Methods**		**EFICAz**	**ModEnzA(Tier I)**	**ModEnzA(Tier I+II)**
**Annotation Benchmark**	**KEGG**			
E. coli				
Sequences	994	856 (1051)	892(1021)	919 (1082)
**Sensitivity**		**86.11**	**89.73**	**92.45**
**Specificity**		**81.44**	**87.36**	**84.93**
EC numbers	750	647 (728)	648 (697)	683 (775)
**Sensitivity**		**86.26**	**86.40**	**91.06**
**Specificity**		**88.87**	**92.96**	**88.12**
B. aphidicola				
Sequences	275	258 (273)	262 (271)	263 (273)
**Sensitivity**		**93.81**	**95.27**	**95.63**
**Specificity**		**94.50**	**96.67**	**96.33**
EC numbers	248	227 (238)	220 (229)	220 (233)
**Sensitivity**		**91.53**	**88.70**	**88.70**
**Specificity**		**95.37**	**96.06**	**94.42**
M. pneumoniae				

Sequences	132	112 (149)	114 (139)	114 (139)
**Sensitivity**		**84.84**	**86.36**	**86.36**
**Specificity**		**75.16**	**82.01**	**82.01**
EC numbers	114	91(122)	102 (122)	102 (122)
**Sensitivity**		**79.82**	**89.47**	**89.47**
**Specificity**		**74.59**	**83.60**	**83.60**
P. falciparum				
Sequences	539	296 (480)	321 (415)	327 (431)
**Sensitivity**		**54.91**	**59.55**	**60.66**
**Specificity**		**61.66**	**77.34**	**75.87**
EC numbers	299	186 (247)	207 (234)	210 (242)
**Sensitivity**		**62.20**	**69.23**	**70.23**
**Specificity**		**75.30**	**88.46**	**86.77**

Numbers within parentheses indicate the total number of sequences or EC numbers identified by each method

Figure SF1 **Six representatives of the Alpha/Beta Hydrolase SCOP fold**

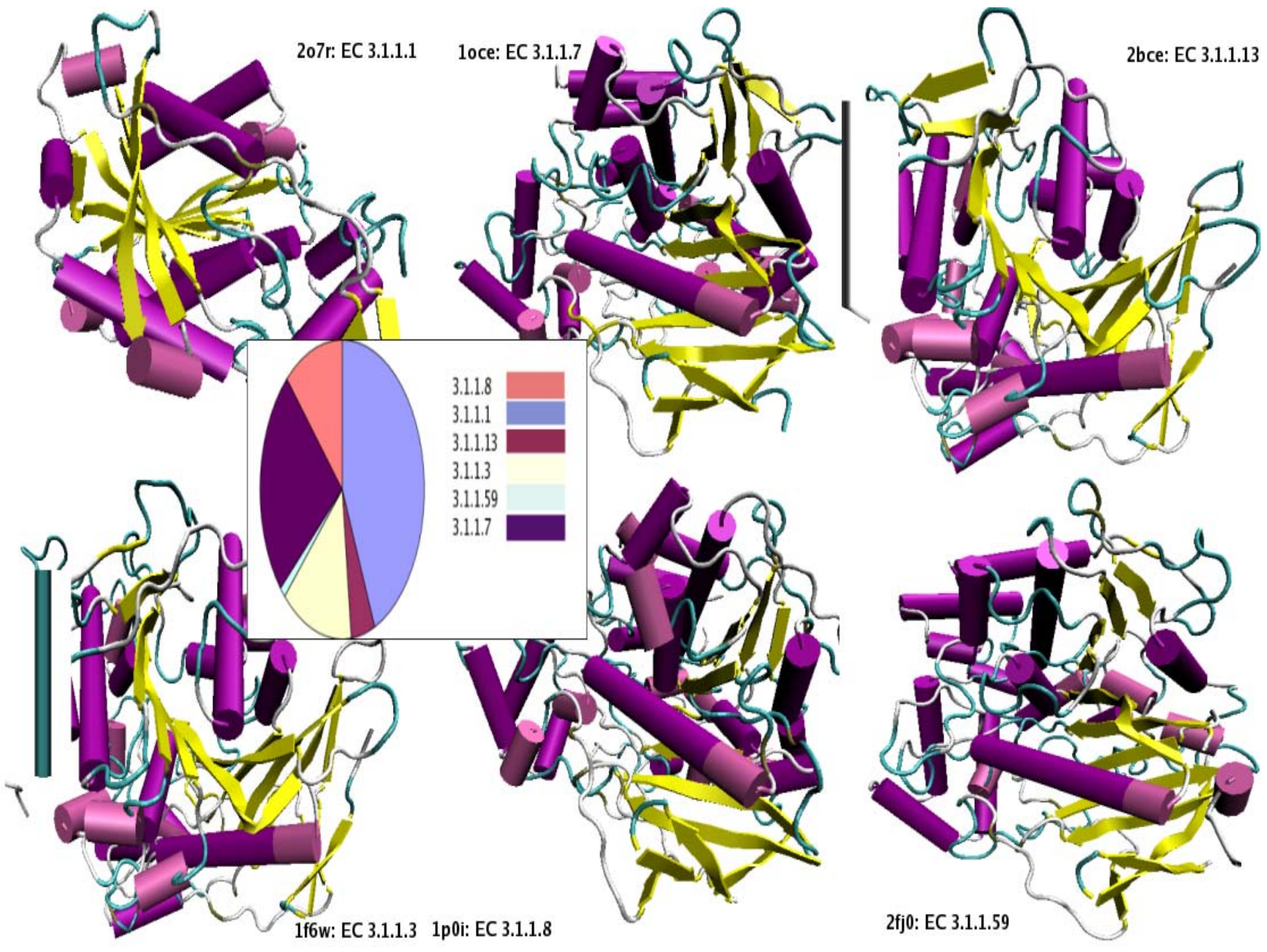

Swiss-Prot sequences from different EC groups selected as False Positives by the HMM profile of EC 3.1.1.8 are shown as inset

Figure SF2 The discriminating potential of the Modenza profiles (the maximum Matthew's Correlation Coefficient value) plotted as a function of the number of training sequences in the subgroup cluster

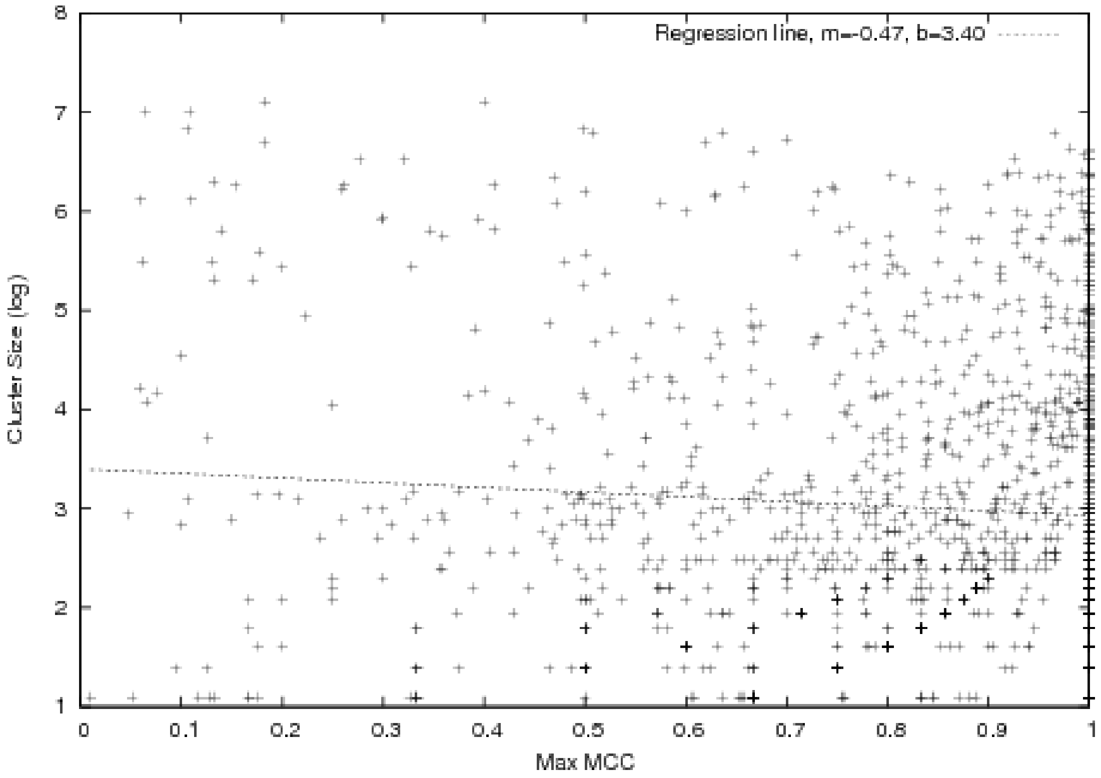

Supplement: Supplementary file 1 — Supplementary data includes 3 tables and 2 figures related to the paper. Table ST1 gives the EC numbers and functional description of some enzymes belonging to the Alpha/Beta Hydrolase fold. Table ST2 provides the correspondence between the subclusters or subgroups of the ModEnzA profile for EC 2.7.7.6 (DNA-directed RNA polymerase) and its structural subunits. Table ST3 shows the comparison between ModEnzA and EFICAz for enzyme identification from 4 completely sequenced genomes using the KEGG annotations for these genomes as the benchmark. Figure SF1 shows the structural similarity in the folds of six enzymes belonging to the Alpha/Beta hydrolase fold. The inset shows the proportion of False Positives selected from the EC groups by the HMM profile of EC 3.1.1.8. Figure SF2 is the plot of the cluster-size of the training sequences and their discriminative potential as indicated by their Average Matthews Correlation Co-efficient (MCC) values. [file 743782.f1.pdf]
